# Antibacterial Activity and Mechanism of Linalool against *Shewanella putrefaciens*

**DOI:** 10.3390/molecules26010245

**Published:** 2021-01-05

**Authors:** Fengyu Guo, Qiong Liang, Ming Zhang, Wenxue Chen, Haiming Chen, Yonghuan Yun, Qiuping Zhong, Weijun Chen

**Affiliations:** 1 College of Food Science and Technology, Hainan University, Haikou 570228, China; Guofengyu1116@163.com (F.G.); lq16448794@163.com (Q.L.); zhangming-1223@163.com (M.Z.); hnchwx@163.com (W.C.); hmchen168@126.com (H.C.); yunyonghuan@foxmail.com (Y.Y.); 2 Key Laboratory of Food Nutrition and Functional Food of Hainan Province, Haikou 570228, China; 3 Hainan Provincial Engineering Research Center of Aquatic Resources Efficient Utilization in the South China Sea, Haikou 570228, China

**Keywords:** linalool, antibacterial mechanism, *Shewanella putrefaciens*, metabolomics

## Abstract

The demand for reduced chemical preservative usage is currently growing, and natural preservatives are being developed to protect seafood. With its excellent antibacterial properties, linalool has been utilized widely in industries. However, its antibacterial mechanisms remain poorly studied. Here, untargeted metabolomics was applied to explore the mechanism of *Shewanella putrefaciens* cells treated with linalool. Results showed that linalool exhibited remarkable antibacterial activity against *S. putrefaciens*, with 1.5 µL/mL minimum inhibitory concentration (MIC). The growth of *S. putrefaciens* was suppressed completely at 1/2 MIC and 1 MIC levels. Linalool treatment reduced the membrane potential (MP); caused the leakage of alkaline phosphatase (AKP); and released the DNA, RNA, and proteins of *S. putrefaciens*, thus destroying the cell structure and expelling the cytoplasmic content. A total of 170 differential metabolites (DMs) were screened using metabolomics analysis, among which 81 species were upregulated and 89 species were downregulated after linalool treatment. These DMs are closely related to the tricarboxylic acid (TCA) cycle, glycolysis, amino acid metabolism, pantothenate and CoA biosynthesis, aminoacyl-tRNA biosynthesis, and glycerophospholipid metabolism. In addition, linalool substantially affected the activity of key enzymes, such as succinate dehydrogenase (SDH), pyruvate kinase (PK), ATPase, and respiratory chain dehydrogenase. The results provided some insights into the antibacterial mechanism of linalool against *S. putrefaciens* and are important for the development and application of linalool in seafood preservation.

## 1. Introduction

Linalool (C_10_H_18_O), also known as 3,7-dimethyl-1,6-octadien-3-ol, is widely found in the essential oils (EOs) extracted from more than 200 plants worldwide, such as green huajiao EOs (28.2% linalool), *Forsythia koreana* leaf EOs (10.68% linalool), and pine needle EOs (24.47% linalool) [1,2,3]. This monoterpene alcohol poses antioxidant, anti-inflammatory, and anticancer activities [4] and exhibits antibacterial activity against *Candida albicans* NCPF 3179, *Staphylococcus aureus* NCTC 10788, *Pseudomonas aeruginosa* NCTC 12924, *Aspergillus brasiliensis* NCPF 2275, and *Escherichia coli* NCTC 12923 [4].

*Shewanella putrefaciens,* the Gram-negative motile bacteria belonging to the family Shewanellaceae, is a seafood spoilage organism (SSO) for cryopreserved fresh fish, that is *S. putrefaciens* is still a great threat to seafood even under cold storage condition [5]. This opportunistic bacteria is also associated with various clinically important infections and diseases, especially in the tropics and during summer months in temperate zones [6]. Adding preservatives to inhibit the growth of putrefying bacteria has become a feasible method. At present, research on the inhibition of putrid bacteria mainly focuses on the combination of antibacterial substances and other materials to improve antibacterial activity. For example, the combination of chitosan and essential oil can effectively inhibit *S. putrefaciens* and extend the shelf life of rainbow trout fillets [7]. The combination of cinnamaldehyde and *Ferulago angulata* essential oil can inhibit the growth of *S. putrefaciens* and thus prolong the shelf life of olive flounder fillets [8].

Metabolomics is the comprehensive analysis of a set of metabolites, the usual end products of biochemical processes and the results of environmental and genetic interactions in a given biological system [9,10], and is a good choice to explore the internal actions of bacteria. This method revealed the synergistic action of electrolyzed water and mild heat for the enhanced microbial inactivation of *Escherichia coli* O157:H7 [11]. Li et al. [12] combined metabolomics and proteomics to study the mechanism of *E. coli* under ciprofloxacin stress.

To the authors’ knowledge, the mechanism underlying the antibacterial activity of linalool against *S. putrefaciens* is poorly understood. This study aimed to investigate the antibacterial property of linalool by adopting the metabolomic approach to provide fundamental information.

## 2. Results

### 2.1. Evaluation of Antibacterial Activity

**Figure 1:**
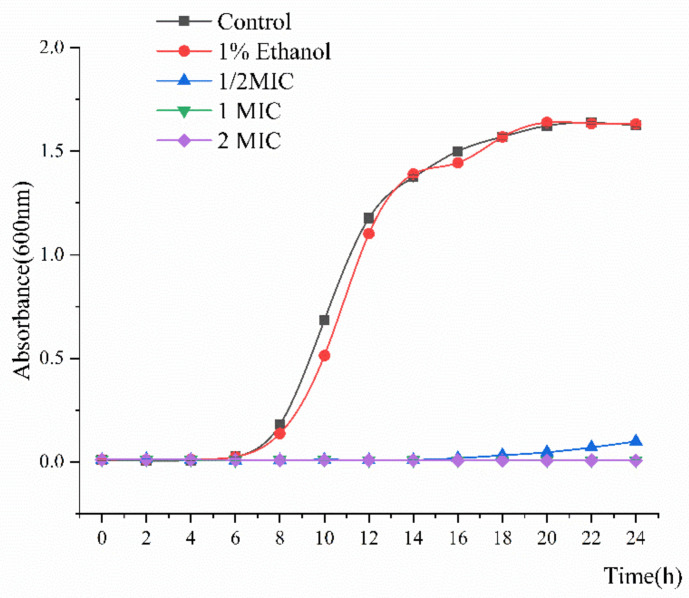
Growth curve of *Shewanella putrefaciens* treated with linalool.

Double dilution revealed that the minimum inhibitory concentration (MIC) of linalool against *S. putrefaciens* was 0.15% *v*/*v*. As shown in Figure 1, the growth curves of *S. putrefaciens* generally followed the model S-shaped growth curve and then reached the logarithmic phase after 5 h and the stable stage at 18 h. However, the growth of *S. putrefaciens* treated with linalool was completely inhibited, indicating the good inhibitory effect of the latter on the former.

### 2.2. Cell Wall Permeability

**Figure 2:**
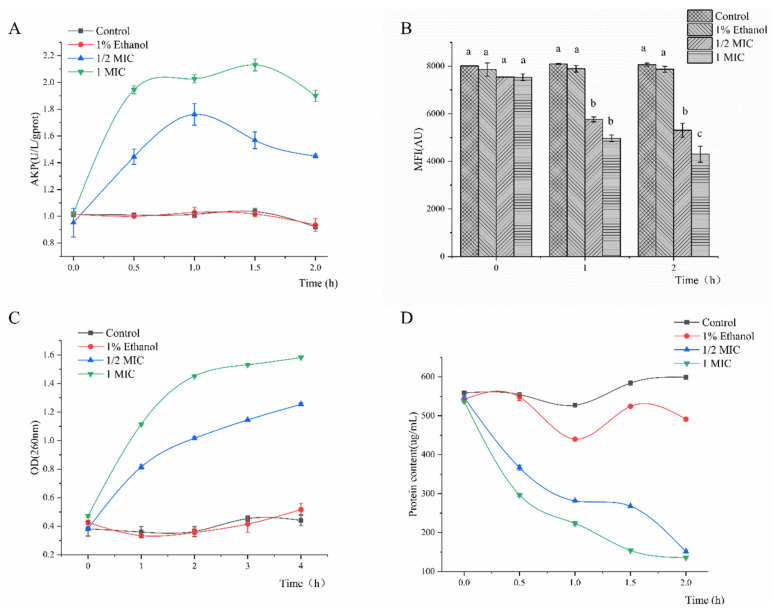
Cell wall permeability (**A**), membrane potential (MP) (**B**), DNA and RNA (**C**), and protein (**D**) of *S. putrefaciens* treated with linalool.

The alkaline phosphatase (AKP) changes in all samples are presented in Figure 2A. The AKP concentration in the suspension of all tested strains treated with linalool (1/2 MIC and MIC) increased remarkably within 2 h. The AKP leakage amount reached a maximum leakage of 2.13 U/L/g protein within 1.5 h. Compared with 1/2 MIC linalool, the 1 MIC group was more effective in increasing the concentration of AKP in the cell suspension, suggesting that high linalool concentrations (MIC) can destroy bacterial cell walls and enhance the membrane leakage of AKP.

### 2.3. Membrane Potential (MP)

Structural damage of the cell membrane may change the MP. Rhodamine 123 is a cationic fluorescent dye that can penetrate the cell membrane. Therefore, its mean fluorescence intensity (MFI) can express the magnitude of MP [13]. The results for MP are shown in Figure 2B. Compared with the control, the bacterial cells treated with linalool showed a significant decrease in MFI at 29.58% for 1/2 MIC group and 42.86% for 1 MIC group after 2 h. Moreover, the fluorescence intensity of *S. putrefaciens* decreased with the increasing linalool concentration, revealing that linalool significantly depolarized the cytoplasmic membrane. Some antibacterial constituents of essential oils can cause cell membrane depolarization and damage [14,15]. These results showed that the MFI was reduced significantly after treatment with linalool, indicating cell membrane damage or apoptosis.

### 2.4. Effect of Linalool on Macromolecules

Cell membrane integrity can be conjectured by measuring the alterations in macromolecules, including DNA and RNA, in the culture medium following linalool addition [15]. Figure 2C shows the results for *S. putrefaciens* treated with different linalool concentrations for 4 h. Compared with those in the control group, a huge change in the DNA and RNA contents in culture medium was observed following the treatment with linalool. The concentration of macromolecules increased by 3.21 and 3.36 times in suspensions treated with 1/2 MIC and 1 MIC linalool, respectively.

### 2.5. Effect of Linalool on Proteins

Proteins can be found throughout the membrane and cytoplasm of bacterial cells and cannot be detected outside the cell. Hence, the leakage quantity of intracellular proteins can be used to evaluate membrane integrity. Following treatment with linalool for 2 h, the release of protein was increased by 72.32% and 74.72% (*p* < 0.05) compared with that of the control group (Figure 2D), revealing that linalool affected the membrane integrity. Cytoplasmic membranes can be irreversibly damaged by treatments with some antibacterial components, leading to the losses of cell constituents such as protein and some essential molecules and even to cell death [16,17]. The release of macromolecules, including DNA, RNA and protein, further confirms the effect of linalool on cell membrane integrity.

### 2.6. Global Analysis of Metabolomic Response

**Figure 3:**
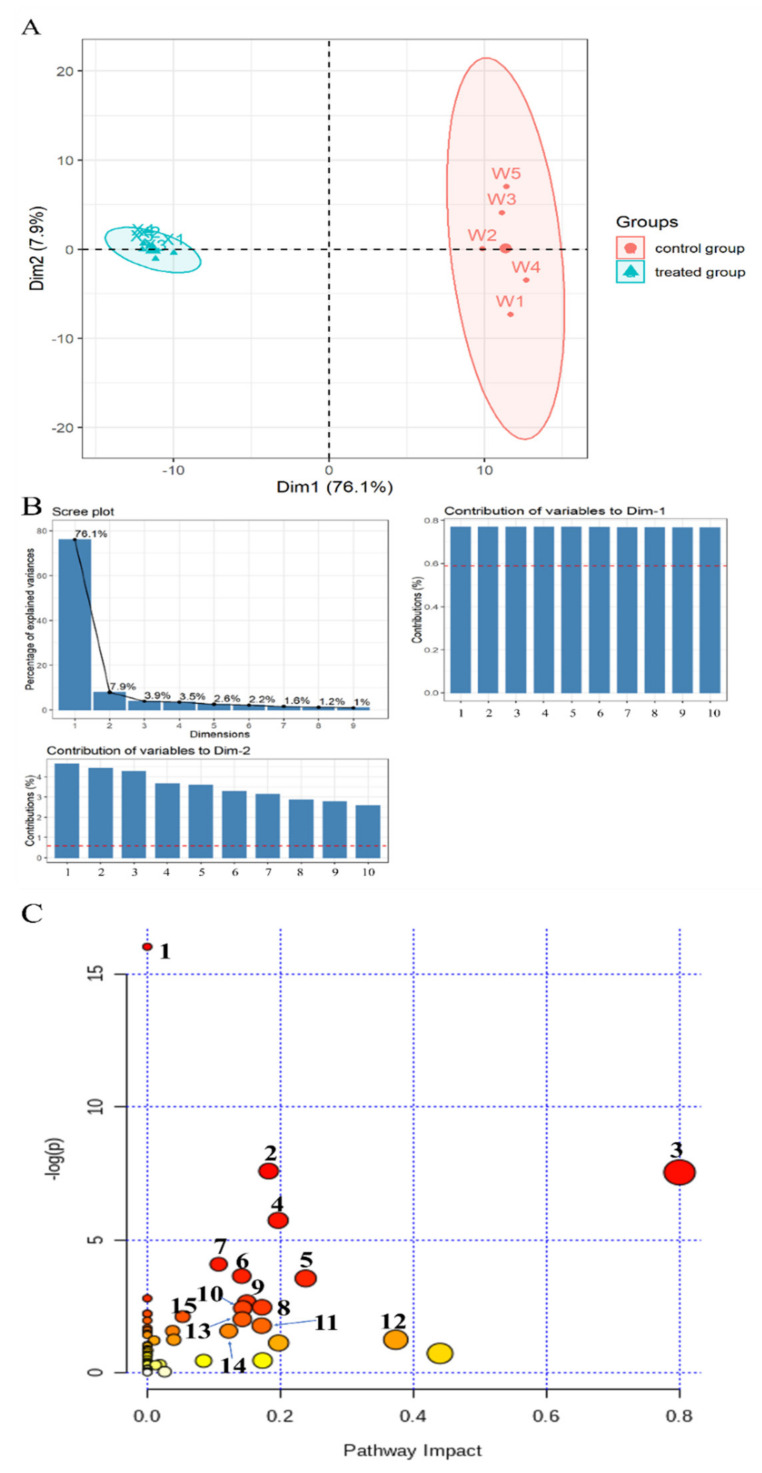
Multivariate cluster analyses of metabolite profiles of treated and control *S. putrefaciens.* (**A**) PCA score plot. (**B**) Scree plot and load plot. Note: Contribution of variables to Dim-2: 1. 4-Guanidinobutanoic; 2. Uracil; 3. Uridine; 4. L-Serine; 5. Ribothymidine; 6. Symmetric dimethylarginine; 7. Amma-glutamyl-L-isoleucine; 8. Oxoglutaric acid; 9. Glutathione; 10. Alanylleucineine. Contribution of variables to Dim-1: 1. 13(S)-HpOTrE; 2. ar-Artemisene; 3. Oleic acid; 4. 13S-HpOTrE(gamma); 5. Linoleic acid; 6. 1-[(5-Oxidanidyl-5-oxidanylidene-L-norvalyl) oxy]octadecane; 7. 24-Oxo-1 alpha, 23, 25-trihydroxyvitamin D3; 8. Octadecanedioic acid; 9. Tert-Butyl 3-amino-1,4,6,7-tetrahydro-5H-pyrazolo[4,3-c]pyridine-5-carboxylate; 10. Glutamylglutamine. (**C**) Kyoto Encyclopedia of Genes and Genomes (KEGG) enrichment of significantly differential metabolites (DMs) with *p* < 0.05 and |log2 (fold change) | > 1. Note: 1. Aminoacyl-tRNA biosynthesis; 2. Arginine biosynthesis; 3. Alanine, aspartate, and glutamate metabolism; 4. Arginine and proline metabolism; 5. Butanoate metabolism; 6. Glycine, serine, and threonine metabolism; 7. Valine, leucine, and isoleucine biosynthesis; 8. D-Glutamine and D-glutamate metabolism; 9. Purine metabolism; 10. Pantothenate and CoA biosynthesis; 11. Citrate cycle (TCA cycle); 12. Pyruvate metabolism; 13. Streptomycin biosynthesis; 14. Glycerophospholipid metabolism; 15. Glyoxylate and dicarboxylate metabolism.

The metabolomics profile of *S. putrefaciens* treated with linalool was determined by LC-MS analysis, which is appropriate for detecting metabolites with low molecular weight. In this method, metabolites with only C, H, and O are detected by the negative ion mode, whereas those also containing N are ionized preferentially in the positive ion mode. Therefore, ionization should be conducted in positive and negative ways to enhance the coverage of metabolomics. Multivariate statistical analysis was also performed on the identified compounds to evaluate the effect of linalool treatment on metabolites. PCA results (Figure 3A) showed that dimensions 1 (Dim-1: 76.1%) and 2 (Dim-2: 7.9%) could explain the cumulative variance ratio of 84%, and the load plot (Figure 3B) displayed the top 10 metabolites with the highest contribution of the first two dimensions. PCA score chart revealed that the first two dimensions in the linalool-treated group and the untreated control group could be completely separated, and Dim-1 could be well distinguished. The 170 kinds of differential metabolites (DMs) were screened according to the *p* value (*p* < 0.05) and fold change (| log_2_ (Fold change) | > 1.5), among which 81 species were upregulated and 89 species were downregulated after treatment with linalool (Supplementary Figure S1). Enrichment analysis of the metabolite pathway (Figure 3C) indicated that the DMs were mostly related to aminoacyl-tRNA biosynthesis, amino acids metabolism, TCA cycle, glycolysis, pantothenate and coenzyme A (CoA) biosynthesis, and lipid metabolism.

### 2.7. Activity of Key Enzymes and Respiratory Metabolism

**Figure 4:**
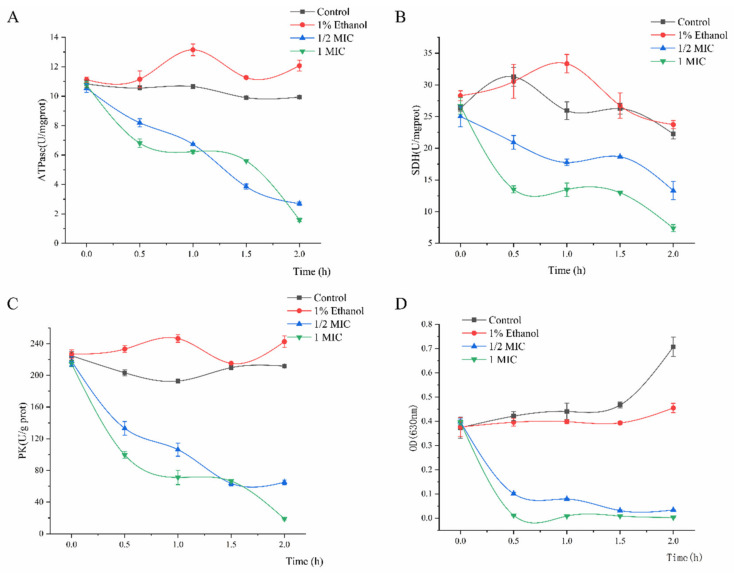
ATPase (**A**), succinate dehydrogenase (SDH) (**B**), pyruvate kinase (PK) (**C**), and respiratory chain dehydrogenase (**D**) activity of *S. putrefaciens* treated with linalool.

ATPase provides cofactors and energy for cells by catalyzing ATP to ADP [18]. A decrease in ATPase activity may hinder carbohydrate metabolism, thus adversely affecting cell growth [19]. Figure 4A shows that compared with that of the control (9.94 U/mg protein), the ATPase activity decreased to 2.69 U/mg protein in the 1/2 MIC group and 1.59 U/mg protein in the 1 MIC group after linalool treatment. Succinate dehydrogenase (SDH) is the only multi-subunit enzyme integrated into the mitochondrial membrane to participate in the oxidative phosphorylation of the cell membrane [20]. The enzyme is associated with respiratory metabolism and oxidative phosphorylation and, therefore, is a representative of mitochondrial enzymes [18]. The SDH values of the treated groups were 13.31 and 7.39 U/mg protein when incubated with linalool at 1/2 MIC and 1 MIC for 2 h, respectively, and those of the control groups were 22.26 and 23.73 U/mg protein. Pyruvate kinase (PK) is one of the major rate-limiting enzymes in glycolysis and converts phosphoenolpyruvate and ADP into ATP and pyruvate [21]. Figure 4C shows that compared with that of the control (211.75 U/g protein), the PK activity decreased to 18.94 U/g protein in the 1 MIC group and 65.04 U/g protein in the 1/2 MIC group after linalool treatment. Iodonitrotetrazolium formazan (INT) can be converted to water-insoluble and dark red (INF) by the bacterial respiratory chain dehydrogenase. INT has an absorption peak at 630 nm, and its decreasing spectrophotometric value can thus be used to assess the loss of respiratory chain enzymatic activity [22,23]. The results showed that linalool treatment almost completely inhibited the respiratory chain dehydrogenase activity of *S. putrefaciens* after 0.5 h in the 1/2 MIC and 1 MIC groups (Figure 4D).

## 3. Discussion

Linalool exhibited remarkable antibacterial activity against *S. putrefaciens*. MIC and growth curve analyses indicated that linalool had strong and consistent inhibiting effects against *S. putrefaciens* (Figure 1). In normal cells, AKP mainly exists between the cell wall and the membrane and is usually not detected outside the cell [13,24]. Figure 2A shows that the content of AKP increases outside the cell, indicating that linalool modifies the membrane structure. As a component of proton dynamics, MP is the potential difference between the inside and outside of biological cells. When the external environment of the cell changes, the ion concentration on opposite sides of the cell membrane is altered, and the cell membrane depolarizes, thus changing the MP and affecting the cell metabolism [13,14]. The results clearly showed that linalool treatment could lead to cell depolarization and affect ATP production and cellular metabolic activity (Figure 2B). Intracellular macromolecules were selected as another aspect to elucidate the antimicrobial action because the integrity of the plasma membrane is a key factor in bacterial growth [15]. The findings showed that the intracellular leakage of macromolecules increased with linalool addition (Figure 2C,D). Therefore, linalool may act on the cell membrane and affect the membrane integrity; thus, nucleic acids and proteins are released through the faulty membrane, and the intracellular leakage of macromolecules is increased [25].

Metabonomic analysis showed that linalool treatment seriously affected the following metabolic pathways of *S. putrefaciens*: (i) amino acid metabolism, (ii) carbohydrate metabolism, (iii) lipid metabolism, (iv) others.

**Figure 5:**
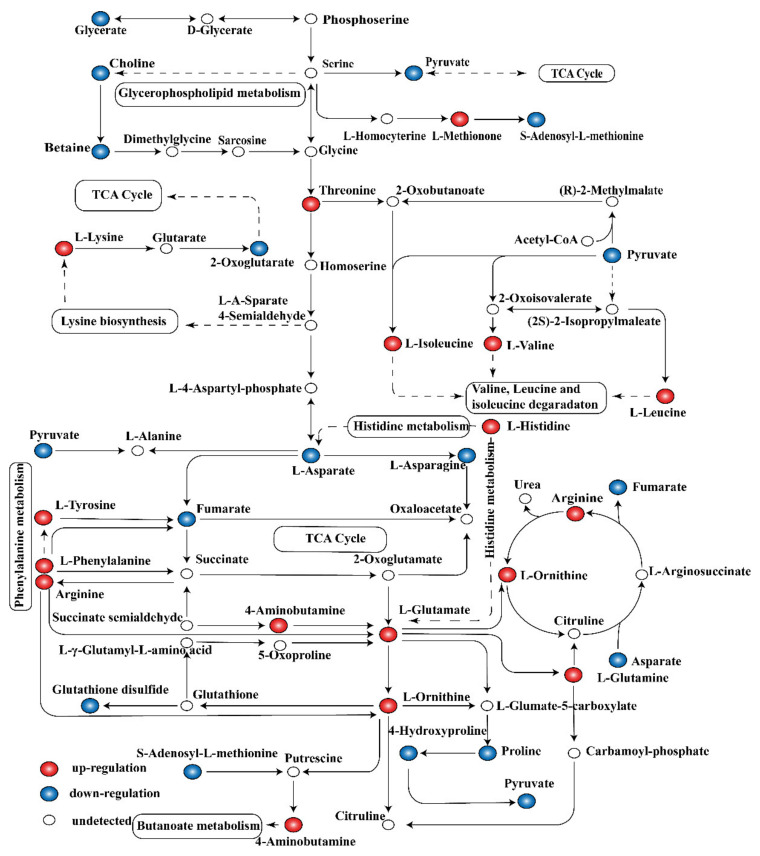
Pathway analysis of differential metabolites (DMs) related to amino acid metabolism.

(i) Amino acid metabolism. In addition to damaging the cellular structure, the release of linalool-treated intracellular contents may lead to a range of metabolic disorders. Bacteria are a complex system with high capacity and flexibility to adapt to various environmental conditions by adjusting their overall metabolic capacity for optimal growth [26]. Owing to its lack of amino acids, *S. putrefaciens* regulates a set of pathways related to amino acid synthesis and metabolism. Bacterial cells exposed to stress typically activate some amino acids biosynthetic pathways to survive [27]. In the case of amino acid metabolism disorder, some amino acid synthesis pathways are activated. DM results also indicate the existence of various amino acid abnormalities. Pathway analysis of DMs related to amino acid metabolism showed that 13 kinds of amino acids in the treatment group were significantly upregulated, whereas three kinds of amino acids were significantly downregulated (Figure 5). This finding indicated disturbance on the synthesis and metabolism pathway of various amino acids.

Peptidoglycan is the main component of the cell envelopes of almost all bacteria, and its structure can act as a selective sieve for molecules from the external environment. As essential components of cell wall peptidoglycan, glutamine and L-lysine are some of the earliest cellular components that encounter extracellular stress [28,29]. When stimulated by the external environment (for example, by linalool), bacteria should maintain the structural integrity of their cell wall. Therefore, the increase in glutamate and lysine in bacterial cells may be used for remodeling the cell wall composition and structure. Similar to the decrease in cell alkaline phosphatase activity, this finding indicates that the integrity of the cell wall is destroyed.

*Acinetobacter baumannii* induces several proteins related to histidine metabolism in the biofilm state, and its biofilm formation is thus triggered by L-histidine metabolism [30]. Another study on the metabolic state of biofilm showed that 12 kinds of proteins related to histidine metabolism were upregulated in the biofilm state [31]. Methionine metabolism regulates the quorum sensing system LuxS regulation in *Streptococcus mutans* and is involved in the bacterial biofilm structural phenotype [32]. In the present study, the upregulation of histidine and methionine may be due to the structure damage of the biofilm caused by linalool treatment, thus stimulating a series of amino acid regulations. This result is also consistent with previous findings on membrane permeability.

Glutamate is an amino donor substrate for all amino transferases in lactic acid bacteria and mediates amino acid metabolism [33]. Most bacteria can convert aspartate to diaminopimelate and lysine, their main sources of lysine [34,35]. As shown in Figure 5, the metabolism of L-lysine and L-aspartate were significantly affected. The metabolic level of L-lysine was upregulated, whereas that of L-aspartate was downregulated. The increased concentrations of L-glutamate, L-glutamine, L-arginine, and L-aspartate contribute to the maintenance of microbial pH homeostasis by consuming H^+^ and producing ATP and NH_3_ [36,37].

As energy-producing substrates and other amino acid synthesis precursors, L-valine, L-leucine, and L-isoleucine play an important role in cell metabolism [38]. These substances regulate proteolysis and act as nitrogen and carbon skeleton donors for the synthesis of other amino acids and the maintenance of their intracellular levels [20,33]. *E. coli* cells can achieve ethanol tolerance by enhancing their amino acid biosynthesis pathways (tryptophan, histidine, valine, leucine, and isoleucine) [27].

**Figure 6:**
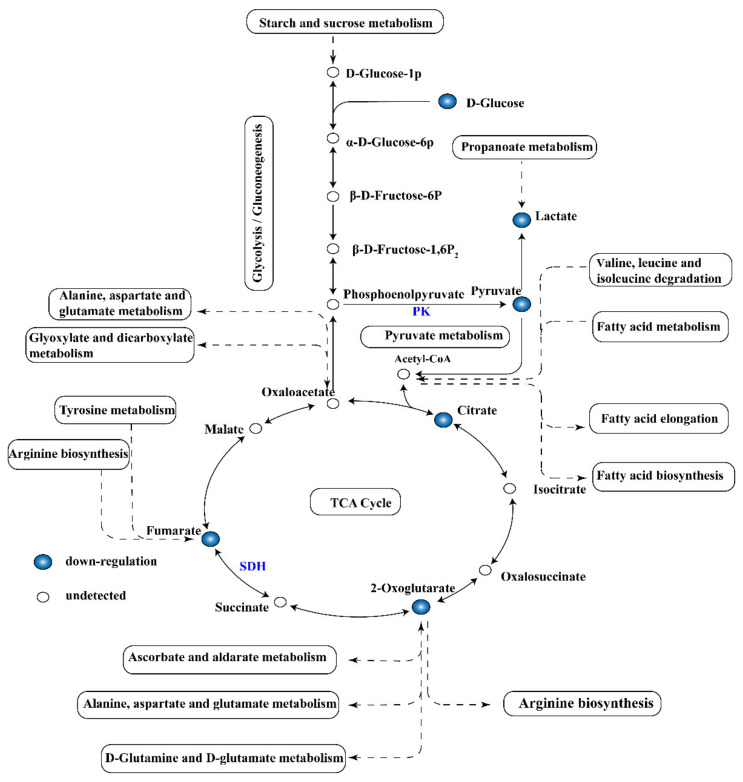
Pathway analysis of DMs related to carbohydrate metabolism.

**Figure 7:**
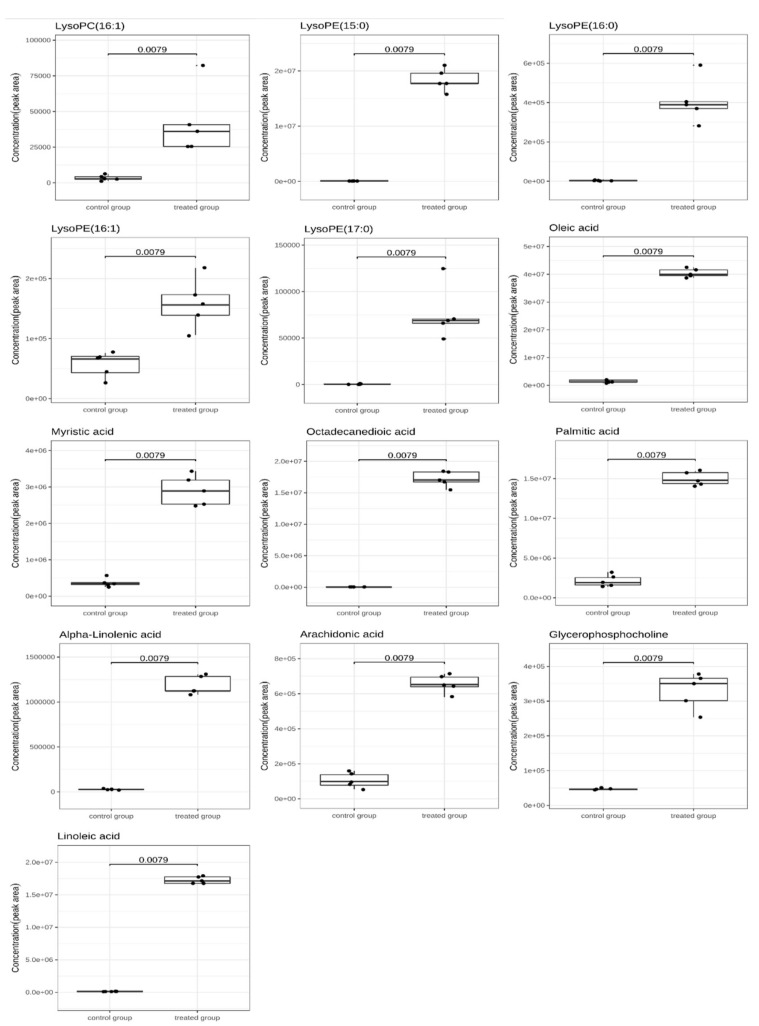
Boxplots showing DMs in lipid metabolism pathways. The horizontal line in the box represents the overall average. The *Y*-axis shows normalized, log-converted, and scaled peak areas. The samples were represented by dots, and the horizontal line in the box is the group average.

Proline is a well-known osmotic regulator and protein stabilizer that protects cells from toxic environments such as salt stress and organic solvent stress [39]. In this study, proline biosynthesis was enhanced, suggesting that these protective osmotic agents protect cells from linalool stress. Amino acid accumulation can enhance protein biosynthesis by replacing damaged proteins and synthesizing stress-related proteins [40]. Thus, the accumulation of multiple amino acids may be ascribed to linalool treatment activating a potential mechanism for protein synthesis in *S. putrefaciens*. In summary, linalool treatment results in the disorder of amino acid metabolism, such as the damaged structure of cell wall and membrane or the abnormal protein synthesis and osmotic regulation.

(ii) Carbohydrate metabolism. Under various environmental stress conditions, microorganisms generally reduce the central energy metabolism to save energy. Glycolysis, the TCA cycle, and the electron transfer chain are important ways to provide energy for cell activity. Hence, the metabolites are usually adversely affected by the environment. The TCA cycle is the main central pathway for most metabolic pathways and provides precursors for metabolic processes such as the oxidative decomposition of carbohydrates, fatty acids, and amino acids (Figure 5). In this study, metabolomics analysis revealed that metabolites such as pyruvate, citrate, fumarate, lactate, and 2-oxoglutarate involved in glycolysis and TCA cycles were downregulated (Figure 6). Therefore, the activity of several key enzymes in glycolysis and TCA cycles were analyzed, and the respiratory chain dehydrogenase and cellular metabolic activities were examined to verify the results of metabonomic analysis (Figure 4). Figure 4A shows that linalool disrupted the ATPase activity, thereby interfering with energy formation and affecting the TCA cycle and glycolysis pathways. This result may be due to the destruction of membrane structure mediated by antimicrobial substances, leading to ATP degradation, increased membrane permeability, and proton (H^+^) transformation [20]. As shown in Figure 4C, linalool treatment inhibited PK activity and thus reduced pyruvate and ATP production. This finding also confirms the decrease in pyruvate content as observed in metabolomics analysis. Pyruvate produced from glycolysis is decarboxylated to acetyl-CoA and then enters the TCA cycle, which is the starting point of the TCA cycle [41]. Succinic acid is converted from succinyl-CoA and then to fumarate under SDH catalysis to produce FADH_2_ [41]. SDH plays a crucial role in the TCA cycle and in the complex II of the mitochondrial electron transport chain and is responsible for transferring electrons from succinate to ubiquinone (coenzyme Q) [42]. A significant reduction in SDH was observed in *S. putrefaciens* treated with linalool (Figure 4B), thus confirming the downregulation of fumarate content in metabolomics. The downregulation of fumarate and SDH enzyme activity may prevent the electron transfer of FADH_2_ and ATP production. Metabolic respiration is the main energy production process for biological growth and, thus, is one of the most common targets of antimicrobial drugs [43]. The results of activity analysis for several key enzymes were consistent with this metabolomics analysis. In addition, the decreased respiratory chain dehydrogenase activity indicated that the bacterial energy metabolic system is damaged. Therefore, carbohydrate metabolism pathways are affected by linalool stress, and the disorder of energy metabolism is a part of linalool’s activity against *S. putrefaciens*.

(iii) Lipid metabolism. Bacteria respond to environmental stress by altering the fluidity of the membrane through changes in the ratio of C16 and C18 fatty acids and the ratio of saturated to unsaturated fatty acids [40,44]. Metabolomics showed the upregulated contents of linolenic acid, linoleic acid, oleic acid, arachidonic acid, myristic acid, octadecanedioic acid, and palmitic acid (Figure 7). The high saturated fatty acid ratio indicates low cell membrane fluidity, which leads to the inhibited entry of acetoin into the cytoplasm and enhanced cell tolerance to acetoin stress [39]. Luo et al. suggested that the high content of unsaturated fatty acids can maintain the original flow of the cell membrane and resist the increase in membrane permeability [45]. In the present study, the content of C16, C18 fatty acids, and saturated and unsaturated fatty acids were upregulated. One possible reason is that different treatments of bacteriostatic substances may affect lipid metabolism to varying degrees. Therefore, the change of the ratio of C16, C18 fatty acids, and saturated and unsaturated fatty acids is one of the mechanisms of *S. putrefaciens* in response to linalool stress, but the specific regulation mode still needs further exploration.

**Figure 8:**
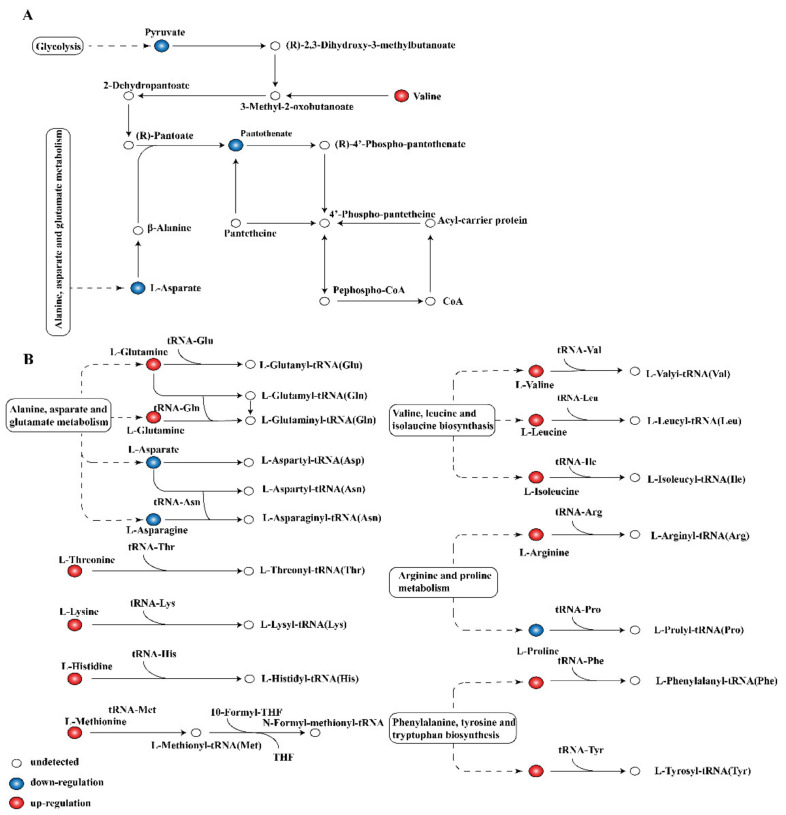
Pathway analysis of DMs related to other metabolic process. (**A**) Pantothenate and CoA biosynthesis, (**B**) aminoacyl–tRNA biosynthesis.

Glycerophosphocholine (GPC) is a hydrolyzed product of two fatty acid chains on phospholipids and plays an important role in phospholipid synthesis and metabolism. This product is an intermediate of phospholipid metabolism and a precursor of phosphatidylcholine and acetylcholine synthesis. GPC can be re-acylated by glycerophosphocholine acyltransferase Gpc1 to produce lysophosphatidylcholine (LPC), which can be transformed into phosphatidylcholine (PC) by lysophospholipid acyltransferase Ale1 [46]. PC and phosphatidyl ethanolamine (PE) are the main phospholipids in the plasma membrane and are important for membrane integrity and fluidity; PE can enhance membrane thickness to improve the resistance of cells to environmental stress [47,48]. In general, linalool may bind to the phospholipids of *S. putrefaciens* membrane, damage the membrane structure, and activate a series of resistance mechanisms to stimulation, thus leading to lipid metabolism disorder.

(iv) Other metabolism pathways. These pathways are critical in cell metabolism because the end products pantothenate and CoA are key cofactors in many pathways involved in phospholipid biosynthesis, fatty acid biosynthesis and degradation, and TCA cycle function [49]. Pantothenate is the precursor of CoA and the repair basis of acyl carrier protein (ACP), which is involved in various metabolic pathways of carbohydrates, fatty acids, and proteins in organisms [20]. The content of pantothenate decreased, and the levels of key precursor related to the biosynthesis of pantothenate and CoA were changed significantly (Figure 8A), indicating that linalool treatment interfered with the synthesis pathway of pantothenate and CoA. Metabolite analysis revealed significant changes in the aminoacyl–tRNA biosynthesis intermediate (Figure 8B). The aminoacyl–tRNA biosynthetic pathway is a part of the protein synthesis translation pathway [50]. Amino acids carried by aminoacyl–tRNA are coupled by peptide bonds in ribosomes in a specific order according to the mRNA sequence, and this step plays a key role in protein biosynthesis [51]. Interference in the aminoacyl–tRNA synthesis pathway affects the related protein synthesis pathway and consequently the cell proliferation and signal transduction [52].

## 4. Materials and Methods

### 4.1. Materials and Chemicals

*S. putrefaciens* strains (ATCC49138) were purchased from Guangdong Microbial Culture Preservation Center (Guangdong, China) and incubated in nutrient broth (NB) medium at 30 °C for 24 h to obtain the log-phase bacteria. Linalool was acquired from Hainan Camphora Biotech Co. Ltd. (Hainan, China). Rhodamine 123, resazurin, and INT were bought from Shanghai Yuanye Bio-technology Co. Ltd. (Shanghai, China). Bicinchoninic acid (BCA), alkaline phosphatase (AKP), succinate dehydrogenase (SDH), pyruvate kinase (PK), and ATPase assay kits were purchased from Nanjing JianCheng Bioengineering Institute (Nanjing, China). All other chemicals were of analytical grade unless otherwise specified. Iron ager medium was obtained from Qingdao Hope Bio-technology Co. Ltd. (Qingdao, China).

### 4.2. MIC Determination

The MIC of linalool was determined through double dilution [53]. Linalool dissolved in alcohol (1%, *v*/*v*) was diluted into a Petri dish containing nutrient agar (NA) solid medium to obtain linalool concentrations of 0.375, 0.75, 1.5, 3, and 6 µL/mL. A sample with sterilized water was used as the blank control, and ethanol (1%) was added to a suspension of the bacteria for the negative control. The Petri dish was then filled with 10^7^ CFU/mL bacterial suspensions (2% of the total culture medium volume) and incubated at 30 °C for 24 h. The MIC value was determined as the lowest concentration of linalool that did not induce bacterial growth.

### 4.3. S. putrefaciens Growth Curve

The antibacterial activity of linalool was assessed through ultraviolet spectrophotometry [53,54]. *S. putrefaciens* (approximately 10^5–6^ CFU/mL) was inoculated into fresh NB medium for 12 h. Linalool dissolved in ethanol (1%, *v*/*v*) was added to the bacterial suspension to reach the final concentration of linalool equal to 1/2 MIC (0.75 µL/mL) and 1 MIC (1.5 µL/mL). NB containing an equal amount of either sterile water or anhydrous ethanol (1%, *v*/*v*) was treated as the blank or negative control. The conical flask with bacterial suspension and linalool was cultured at 150 rpm and 30 °C, and the cell concentration was determined by measuring the OD_600_ nm at 1 h intervals with a UV spectrophotometer (TU1810, Beijing, China).

### 4.4. MP Determination

MP was determined by rhodamine fluorescence with modification [13,14,15]. *S. putrefaciens* were grown to the log-phase in NB, cultured in a rotary shaker (150 rpm) at 30 °C and treated with linalool (final concentration 1/2 MIC and 1 MIC, with ethanol and water as the control group) for 0, 0.5, 1, 1.5, and 2 h. The bacterial cells were centrifuged, washed, and resuspended with phosphate buffer solution (PBS) (0.01 M, pH 7.2), and rhodamine 123 was then added to a final concentration of 2 mg/mL. After the solution stood in the dark for 30 min, the samples were washed and resuspended in PBS (0.01 M, pH 7.2). Finally, fluorescence was immediately measured at the excitation and emission wavelengths of 480 and 530 nm, respectively, by a fluorescence spectrophotometer (WGY-10, Tianjin Gangdong Sci. and Tech. Development Co. LTD, Tianjin, China). Data were expressed by mean fluorescence intensity (MFI).

### 4.5. Alkaline Phosphatase (AKP) Activity Determination

Linalool was added to the log-phase bacteria suspension to obtain the final concentrations of 1/2 MIC and 1 MIC and incubated at 30 °C. The suspension was collected through centrifugation at 30 min intervals, and AKP activity was determined by using an AKP detection kit (Jiancheng Bioengineering Institute, Jiangsu, China) following the manufacturer’s instructions, and AKP was expressed in U/L/g protein.

### 4.6. Loss of DNA and RNA

Log-phase *S. putrefaciens* was obtained through centrifugation (6000 rpm for 10 min) and washed twice with PBS (0.01 M, pH 7.2). The precipitate was further resuspended in PBS and treated with linalool (final concentration 1/2 MIC and 1MIC, with ethanol and water as the control group) for 0, 0.5, 1, 1.5, and 2 h. Following the reaction, the suspensions were centrifuged at 6000 rpm for 10 min at 4 °C. The supernatant was determined by an ultraviolet spectrophotometer (TU1810, Beijing, China) at 260 nm [16,55].

### 4.7. Protein Leakage

Protein leakage was tested by Bicinchoninic acid (BCA) Protein Assay Kit following the manufacturer’s instructions (Jiancheng Bioengineering Institute, Jiangsu, China) [56]. Bacterial cell solutions (approximately 10^7^ CFU/mL) were added with different concentrations of linalool (ethanol and water as the control, 1/2 MIC and 1 MIC levels). After incubation for 0, 0.5, 1, 1.5, and 2 h, the bacterial cells were centrifuged, washed with PBS (0.01 M, pH 7.2), and resuspended to OD _600_ nm = 2.0. Afterward, 1 mL of the bacterial suspension was centrifuged (5000 rpm, 10 min), and the precipitate was washed with PBS (0.01 M, pH 7.2) three times. The prepared bacterial cells were further resuspended in 1 mL of PBS for 10 min ultrasonic processing (power 300W, interval 1.1 s) in an ice bath. Finally, the homogenate was used to determine the protein content and enzyme activity.

### 4.8. Metabolomics Analysis

Sample preparation for metabolomics analysis. Linalool with 1 MIC concentration was added to the log-phase bacterial suspension, and sterile water was used as the control. After being cultured at 30 °C for 1.5 h, the bacterial cells were collected through centrifugation at 1500 rpm for 10 min and washed twice with cold PBS. The trace medium was removed through centrifugation again. The bacterial cells were quenched with liquid nitrogen and stored at −80 °C prior to analysis.

Extraction of intracellular metabolites. In accordance with a previous method [57], intracellular metabolites were extracted from each biological replicate through cold methanol extraction, and five biological replicates were obtained per group. The samples were smashed with a bead grinder (5 m/s, 8 s, two cycles), mixed with 1 mL of 80% methanol, and incubated at a temperature of 4 °C at 1500 rpm for 30 min. The extracted supernatant was first centrifuged at 4 °C for 10 min at 12,000 rpm and then dried using SpeedVac. The dried sample was redissolved in 100 µL of 1% acetonitrile. The supernatant was centrifuged at rpm for 10 min for measurement.

HPLC-ESI/QTOF-MS measurement. The metabolites were quantified using Agilent 1290 II (Agilent, Germany) with ACQUITY UPLC HSS T3 column (1.8 μm, 2.1 × 100 mm). The column oven temperature was maintained at 40 °C, and the auto-sampler temperature was maintained at 4 °C. The samples were eluted on a column with gradient elution separation at 0.35 mL/min using solvents A (0.1% formic acid in water) and B (0.1% formic acid in acetonitrile). The linear gradient program as follows: initial 1% B for 1 min; 1~5 min, increase in B from 1% to 40%; 5~8 min, increase in B from 40% to 50%; 8~10 min, increase in B from 50% to 65%; 10~12 min, increase in B from 65% to 70%; 12~15 min, held at 100% B; and 15~18 min, starting mobile phase 1% B to re-equilibrate the column. The flow rate was 0.35 mL/min, and the injection volume was 2 μL.

MS was performed using 5600 Triple TOF Plus (AB Sciex, Singapore). All analyses were performed in electrospray ionization (ESI±) mode under the following conditions: curtain gas flow rate = 35, spray voltage = 5500 V (positive ion mode), spray voltage = −4500 V (negative ion mode), ion source temperature = 500 °C, spray gas flow rate = 50, and auxiliary gas flow rate = 50. Random sorting of samples was conducted to reduce systematic errors and provide reliable experimental results during instrument detection. Ten samples were tested, and three QC samples were inserted to monitor the stability of the instrument.

MarkerView 1.3 (AB Sciex, Concord, ON, Canada) software was used to determine the peak area, mass, mass-to-charge ratio, and storage time of the original data, and generate 2D data array (filter the isotope peak). Comparison of secondary mass spectrometry data, metabolite database, Human Metabolome Database (HMDB), Metabolite and Tandem MS Database (METLIN), and standard products was performed by PeakView 2.2 (AB Sciex, Concord, ON, Canada) to identify the metabolite ID. The identified ID was assigned to the corresponding ion in the 2D data array of primary mass spectrometry, and the identified metabolomics data were statistically analyzed based on R language. Metabolites with variable importance in the projection fold values (up- or down regulation) larger than 1.5 and with *p* value less than 0.05 were identified as differential metabolites (DMs). Metabolic pathway analysis was performed using the online tool MetaboAnalyst 3.0 (http://www.metaboanalyst.ca/).

### 4.9. Activity Determination for Adenosine Triphosphatase (ATPase), Succinic Acid Dehydrogenase (SDH), and Pyruvate Kinase (PK)

Homogenate was prepared according to 4.7, and its enzyme activity was measured with ATPase, SDH, and PK detection kits (Jiancheng Bioengineering Institute, Jiangsu, China) following the manufacturer’s instructions. Enzyme activity was expressed in U/mg protein or U/g protein.

### 4.10. Activity of Respiratory Chain Dehydrogenase

The enzymatic activity of respiratory chain dehydrogenase was determined by INT indicator with some modifications [23,58]. Log-phase bacterial cells were centrifuged at 4000 r/min for 10 min, washed, and suspended with sterile normal saline. The prepared bacterial suspensions were added with 1/2 MIC and 1 MIC with sterile water and 1% ethanol as the control, cultured for 0, 0.5, 1, 1.5, and 2 h, and centrifuged in low temperature for 10 min at 4000 r/min. The bacterial cells were collected and washed with sterile normal saline three times and then resuspended. In brief, 150 µL of 0.01 mol/L INT was prepared with methanol and water (1:1) and added with 1350 µL of bacterial suspension to reach the final INT concentration of 1 mmol/L. The solution was placed at 30 °C for 30 min and measured at 630 nm. The activity of respiratory chain dehydrogenase was indicated by the absorbance value of 630 nm.

### 4.11. Statistical Analysis

All experiments were performed in triplicate. Data were expressed as the mean ± SD. One-way ANOVA and Duncan’s multiple range tests were used to express the significance of differences (*p* < 0.05) between means.

## 5. Conclusions

UPLC-MS/MS metabolomics system was used to study the inhibitory effect of linalool on *S. putrefaciens* and provide new insights into the inhibitory mechanism of linalool on *S. putrefaciens*. The results showed that linalool treatment could effectively inhibit the growth of *S. putrefaciens*, destroy the structure and function of the cell wall and membrane, induce the leakage of intracellular substances, and cause the dysfunction of energy and metabolism. Metabolomics analysis showed that linalool treatment caused significant changes in 170 metabolites. Pathway analysis for these 170 differential metabolites revealed that many metabolic pathways were disturbed, including amino acid synthesis and metabolism pathway, carbohydrate metabolism pathway, lipid metabolism pathway, pantothenate and CoA pathway, and aminoacyl–tRNA biosynthesis pathway. These results provide new insights and research direction for studying the inhibiting ability of linalool on the metabolic changes of bacteria at the molecular level and serve as a useful guide for the development of food-related bacteriostatic agents by using linalool and other essential oils from plants.

## Data Availability

The data presented in this study are available in article and Supplementary Material.

## Supplementary Materials

The following are available online at, Figure S1: Heatmap.
