# Supplementary material for: Antibacterial Activity and Mechanism of Linalool against Shewanella putrefaciens
Source: Molecules. 2021 Jan 5;26(1):245. doi: 10.3390/molecules26010245 (PMC7796449; doi:10.3390/molecules26010245)
Supplement: Supplementary file 1 [file molecules-26-00245-s001.pdf]

molecules

Supplementary Materials

Antibacterial Activity and Mechanism of Linalool Against *Shewanella putrefaciens*

Fengyu Guo [1,2,3], Qiong Liang [1], Ming Zhang [1], Wenxue Chen [1,2,3], Haiming Chen [1,2,3], Yonghuan Yun [1,2,3], Qiuping Zhong [1,2,3,*] and Weijun Chen [1,2,3,*]

1. College of Food Science and Technology, Hainan University, Haikou 570228, China
2. Key Laboratory of Food Nutrition and Functional Food of Hainan Province, Haikou 570228, China
3. Hainan Provincial Engineering Research Center of Aquatic Resources Efficient Utilization in the South China Sea, Haikou 570228, China

* Correspondence: hainufood88@163.com (Q.Z.), chenwj@nwu.edu.cn (W.C.)

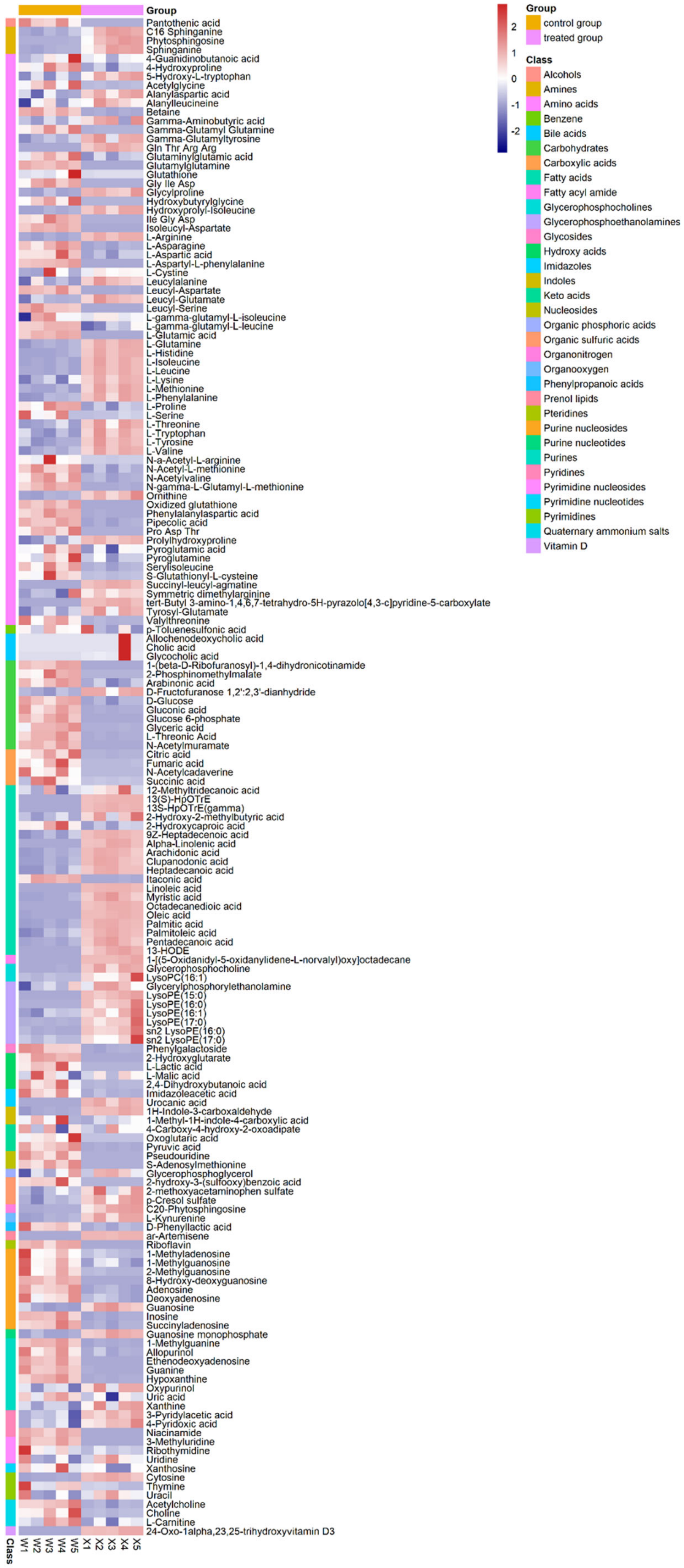
Group
Pantothenic acid
C16 Sphinganine
Phytosphingosine
Sphinganine
4-Guanidinobutanoic acid
4-Hydroxyproline
5-Hydroxy-L-tryptophan
Acetylglycine
Alanylaspartic acid
Alanylleucineine
Betaine
Gamma-Aminobutyric acid
Gamma-Glutamyl Glutamine
Gamma-Glutamyltyrosine
Gln Thr Arg Arg
Glutaminylglutamic acid
Glutamylglutamine
Glutathione
Gly Ile Asp
Glycylproline
Hydroxybutyrylglycine
Hydroxyprolyl-Isoleucine
Ile Gly Asp
Isoleucyl-Aspartate
L-Arginine
L-Asparagine
L-Aspartic acid
L-Aspartyl-L-phenylalanine
L-Cystine
Leucylalanine
Leucyl-Aspartate
Leucyl-Glutamate
Leucyl-Serine
L-gamma-glutamyl-L-isoleucine
L-gamma-glutamyl-L-leucine
L-Glutamic acid
L-Glutamine
L-Histidine
L-Isoleucine
L-Leucine
L-Lysine
L-Methionine
L-Phenylalanine
L-Proline
L-Serine
L-Threonine
L-Tryptophan
L-Tyrosine
L-Valine
N-a-Acetyl-L-arginine
N-Acetyl-L-methionine
N-Acetylvaline
N-gamma-L-Glutamyl-L-methionine
Ornithine
Oxidized glutathione
Phenylalanylaspartic acid
Pipecolic acid
Pro Asp Thr
Prolylhydroxyproline
Pyroglutamic acid
Pyroglutamine
Serylisoleucine
S-Glutathionyl-L-cysteine
Succinyl-leucyl-agmatine
Symmetric dimethylarginine
tert-Butyl 3-amino-1,4,6,7-tetrahydro-5H-pyrazolo[4,3-c]pyridine-5-carboxylate
Tyrosyl-Glutamate
Valylthreonine
p-Toluenesulfonic acid
Allochenodeoxycholic acid
Cholic acid
Glycocholic acid
1-(beta-D-Ribofuranosyl)-1,4-dihydronicotinamide
2-Phosphinomethylmalate
Arabinonic acid
D-Fructofuranose 1,2':2,3'-dianhydride
D-Glucose
Gluconic acid
Glucose 6-phosphate
Glyceric acid
L-Threonic Acid
N-Acetylmuramate
Citric acid
Fumaric acid
N-Acetylcadaverine
Succinic acid
12-Methyltridecanoic acid
13(S)-HpOTrE
13S-HpOTrE(gamma)
2-Hydroxy-2-methylbutyric acid
2-Hydroxycaproic acid
9Z-Heptadecenoic acid
Alpha-Linolenic acid
Arachidonic acid
Clupanodonic acid
Heptadecanoic acid
Itaconic acid
Linoleic acid
Myristic acid
Octadecanedioic acid
Oleic acid
Palmitic acid
Palmitoleic acid
Pentadecanoic acid
13-HODE
1-[(5-Oxidanidyl-5-oxidanylidene-L-norvalyl)oxy]octadecane
Glycerophosphocholine
LysoPC(16:1)
Glycerylphosphorylethanolamine
LysoPE(15:0)
LysoPE(16:0)
LysoPE(16:1)
LysoPE(17:0)
sn2 LysoPE(16:0)
sn2 LysoPE(17:0)
Phenylgalactoside
2-Hydroxyglutarate
L-Lactic acid
L-Malic acid
2,4-Dihydroxybutanoic acid
Imidazoleacetic acid
Urocanic acid
1H-Indole-3-carboxaldehyde
1-Methyl-1H-indole-4-carboxylic acid
4-Carboxy-4-hydroxy-2-oxoadipate
Oxoglutaric acid
Pyruvic acid
Pseudouridine
S-Adenosylmethionine
Glycerophosphoglycerol
2-hydroxy-3-(sulfooxy)benzoic acid
2-methoxyacetaminophen sulfate
p-Cresol sulfate
C20-Phytosphingosine
L-Kynurenine
D-Phenyllactic acid
ar-Artemisene
Riboflavin
1-Methyladenosine
1-Methylguanosine
2-Methylguanosine
8-Hydroxy-deoxyguanosine
Adenosine
Deoxyadenosine
Guanosine
Inosine
Succinyladenosine
Guanosine monophosphate
1-Methylguanine
Allopurinol
Ethenodeoxyadenosine
Guanine
Hypoxanthine
Oxypurinol
Uric acid
Xanthine
3-Pyridylacetic acid
4-Pyridoxic acid
Niacinamide
3-Methyluridine
Ribothymidine
Uridine
Xanthosine
Cytosine
Thymine
Uracil
Acetylcholine
Choline
L-Carnitine
24-Oxo-1alpha,23,25-trihydroxyvitamin D3
Class
W1
W2
W3
W4
W5
X1
X2
X3
X4
X5
2
1
0
-1
-2
Group
control group
treated group
Class
Alcohols
Amines
Amino acids
Benzene
Bile acids
Carbohydrates
Carboxylic acids
Fatty acids
Fatty acyl amide
Glycerophosphocholines
Glycerophosphoethanolamines
Glycosides
Hydroxy acids
Imidazoles
Indoles
Keto acids
Nucleosides
Organic phosphoric acids
Organic sulfuric acids
Organonitrogen
Organooxygen
Phenylpropanoic acids
Prenol lipids
Pteridines
Purine nucleosides
Purine nucleotides
Purines
Pyridines
Pyrimidine nucleosides
Pyrimidine nucleotides
Pyrimidines
Quaternary ammonium salts
Vitamin D

Figure S1
